# Supercritical CO_2_ Treatment to Modify Techno-Functional Properties of Proteins Extracted from Tomato Seeds

**DOI:** 10.3390/foods13071045

**Published:** 2024-03-28

**Authors:** Paola Mateo-Roque, Jocksan I. Morales-Camacho, Guadalupe Janet Jara-Romero, Flor de Fátima Rosas-Cárdenas, Luis Huerta-González, Silvia Luna-Suárez

**Affiliations:** 1 Centro de Investigación en Biotecnología Aplicada, Instituto Politécnico Nacional, Tepetitla 90700, Tlaxcala, Mexico; paomaro9133_@hotmail.com (P.M.-R.); janet_16@live.com.mx (G.J.J.-R.); frosasc@ipn.mx (F.d.F.R.-C.); lhuertag@ipn.mx (L.H.-G.); 2 Departamento de Ingeniería Química, Alimentos y Ambiental, Universidad de las Américas Puebla, San Andrés Cholula 72810, Puebla, Mexico; jocksan.morales@udlap.mx

**Keywords:** tomato seed protein, supercritical CO_2_, structural characterization of proteins, techno-functional properties, FT-IR spectroscopy

## Abstract

Tomato seeds are a rich source of protein that can be utilized for various industrial food purposes. This study delves into the effects of using supercritical CO_2_ (scCO_2_) on the structure and techno-functional properties of proteins extracted from defatted tomato seeds. The defatted meal was obtained using hexane (TSMH) and scCO_2_ (TSMC), and proteins were extracted using water (PEWH and PEWC) and saline solution (PESH and PESC). The results showed that scCO_2_ treatment significantly improved the techno-functional properties of protein extracts, such as oil-holding capacity and foaming capacity (especially for PEWC). Moreover, emulsifying capacity and stability were enhanced for PEWC and PESC, ranging between 4.8 and 46.7% and 11.3 and 96.3%, respectively. This was made possible by the changes in helix structure content induced by scCO_2_ treatment, which increased for PEWC (5.2%) and decreased for PESC (8.0%). Additionally, 2D electrophoresis revealed that scCO_2_ hydrolyzed alkaline proteins in the extracts. These findings demonstrate the potential of scCO_2_ treatment in producing modified proteins for food applications.

## 1. Introduction

Tomato (*Solanum lycopersicum* L.) is the second most important vegetable crop in the world [1]. The annual global tomato production amounts to 182 million tons, and Mexico is a main producer country on the American continent. Tomato is the best-selling Mexican export [2]. Approximately 42.5 million tons of annual production are used for tomato processing in the industry [3]. During tomato processing to produce sausages, puree, or juices, a waste material known as tomato pomace is generated. This material is about 5% by weight of the processed tomatoes [4], which represents 2.15 million tons of waste. Traditionally, such waste is mainly used as animal feed or for composting or is discharged in landfills [5].

Tomato pomace consists mainly of tomato peels, pulp residues, and seeds [4]. Tomato seeds are typically a waste product from the food canning industry and find use in animal feeding [6]. However, when these residues are not utilized, they contribute to disposal problems and environmental pollution [7]. Interestingly, tomato seeds account for approximately 60% of the total waste and are a rich source of proteins [8]. Thus, extracting proteins from tomato seeds presents an alternative solution for the food industry, utilizing an inexpensive resource and could help reduce waste and pollution.

Different studies have reported the techno-functional properties of the proteins found in seeds, such as their ability to absorb water and oil, form gels and foams, and emulsify. These properties are determined by a range of factors, including the size and structure of proteins, their 3D conformation, and their charge distribution. All these factors are also influenced by processing conditions, including temperature, pH, and the solvents used for extraction, among others [9]. Traditionally, oil extraction from meal involves the use of organic solvents under either cold or hot conditions. However, supercritical fluid extraction using CO_2_ (scCO_2_) is an alternative for oil extraction that is considered a green technology due to its harmless and environmentally safe properties, as well as its quick removal from products. The temperature used for scCO_2_ is close to room temperature [10,11], and in order to achieve it, a critical temperature of 31.1 °C and a pressure of 7.38 MPa are necessary; above these conditions, the supercritical state is maintained [12]. However, the latter thermodynamic variable has not been widely studied to observe the effect on the techno-functional properties of proteins [13].

Therefore, the purpose of this study was to obtain protein extracts from defatted tomato seed meal using hexane (TSMH) and supercritical CO_2_ (TSMC) and assess their impacts on the techno-functional properties and structural attributes of the proteins. To achieve this, the water/oil absorption, solubility, foaming capacity, and emulsifying capacity of protein extracts were measured. Additionally, FT-IR spectroscopy was employed to determine their structural characteristics, and protein profile analyses were conducted using SDS-PAGE and 2D-PAGE analysis.

## 2. Materials and Methods

### 2.1. Pomace and Tomato Seed Meals

Fresh tomatoes of the saladette variety were acquired from a local market in Puebla city. Sodium dodecyl sulphate polyacrylamide gel electrophoresis (SDS PAGE) reagents were purchased from Bio-Rad. Analytical-grade NaOH (Meyer, Mexico), NaCl, hexane, and HCl (J. T. Baker, Xalostoc, Mexico) were employed. Distilled water was used as a solvent in all experiments.

A lot of 20 kg of fresh tomatoes was used; the tomatoes were washed, and their peels and seeds were removed manually. The seeds were separated by sedimentation in water, as described by Rossini et al. [7], mixing them with distilled water (1:10), and stirred manually with a spoon for 3 min. Subsequently, the mixture was left to rest for 5 min; during this time, the skin and pulp remained on the surface, and the seeds remained at the bottom of the container. After this time, the water, pulp, and peels were decanted. This procedure was repeated until only the seeds remained. Each fraction of tomato was weighed to determine the amount of pomace, which was calculated by dividing the weight of the pomace by the weight of the fresh tomato and multiplying by 100. Afterward, the seeds were dried at room temperature and ground in a KRUPS GX4100 mill to produce a fine powder (600 ± 50 µm).

### 2.2. Chemical Composition Analysis

The chemical composition of the seeds was determined using the following analytical methods [14]: moisture content (m 934.06 AOAC), ash content (m 930.05 AOAC), total fat content determined by the Soxhlet method (m 903.09 AOAC), total protein evaluated by the Kjeldahl method with a protein conversion factor of 6.25 (m 978. 04 AOAC), and crude fiber content (m 962.09 AOAC). The carbohydrate content was determined by the weight difference.

### 2.3. Tomato Seed Meal Degreasing

Tomato seed meal was divided into two equal parts; one part was defatted using hexane (TSMH), with the defatting process being carried out overnight at room temperature. The meal was mixed with hexane in a 1:10 ratio (solute:solvent) and stirred at 33 rpm in a tubular roller mixer. The sample was then centrifuged at room temperature, with the supernatant being discarded. The solvent residues of the meal were finally evaporated at 40 °C [2] in a rotary evaporator. The other part of tomato seed meal was defatted with scCO_2_ (TSMC); it was obtained using the supercritical fluid extraction equipment that was assembled at the University of the Americas Puebla as previously reported by Conde-Hernández, et al. [15]. The conditions for the extraction process were set based on previous experiments [16]; samples (20 g) of the meal were placed into the extraction cell and subjected to a temperature of 50 ± 0.1 °C and pressure of 24.82 MPa. The CO_2_ volumetric flow rate was set at 130 ±18 mL/min for semi-continuous extraction over 6 h, with a sample (oil extraction) taken every 30 min in a recollection cell that was immersed in a water bath at 8 ± 0.5. Finally, TMSC was recovered and stored at −20 °C in a sealed bag for further use.

### 2.4. Protein Extraction, Yield and Quantitation

To extract the protein, a tomato seed meal sample from TSMH was mixed with water (pH 6.8), as well as with water at two different pH values (8 and 10) adjusted with 0.1 M NaOH and different saline solutions (0.5 and 1 M NaCl). The meal was mixed with the solution in a ratio of 1:10 (solute:solvent) and stirred at 33 rpm using an SRT6 (Stuart) tubular roller mixer at room temperature for one hour. The slurry was centrifuged at 13,000 rpm for 3 min at room temperature. The supernatant was collected, and the protein content was measured by the bicinchoninic acid (BCA) method [17] with bovine serum albumin as the standard.

Protein extraction yield percentage was calculated by dividing the total extracted protein by the total protein found in the raw material (tomato seed meal). Protein extracts were dried at 50 °C in an oven and stored at −80 °C for further assays.

In order to obtain a water-soluble protein extract (PEWC) or a salt-soluble protein extract (PESC) from the tomato seed meal that had been defatted with scCO_2_, the sample was mixed with water at pH 6.8 or with a saline solution (1 M NaCl), respectively. To obtain the water-soluble protein extract (PEWH) or the salt-soluble protein extract (PESH) from tomato seed meal defatted with hexane, the same procedure described above was followed.

### 2.5. Electrophoretic Pattern: SDS-PAGE and 2D PAGE Analysis

Proteins found in tomato seed meal (TSMH and TSMC) and their protein extracts were analyzed using SDS-PAGE under reducing conditions according to Laemmli [18]. A sample from flour or protein extracts (10 mg, dry basis) was weighted and mixed with 500 µL of SDS reducing buffer, and the sample was heated to 95 °C. Then, 5 µL of the prepared sample was loaded on to the gel (14%), and electrophoresis applied using a Mini-PROTEAN tetra cell (Bio-Rad, Hercules, CA, USA). The gel was then stained with a Coomassie Brilliant Blue R-250 solution for 20 min and destained with a 10% acetic acid solution. Finally, the gel was scanned using a ChemiDoc XRS+ System, and “Image lab” software (Version 6.0) was utilized to determine the molecular weight of the proteins, using a standard molecular protein weight marker supplied by Bio-Rad.

Subsequently, 2D PAGE analysis was performed using 7 cm immobilized strips of 3–10 pH gradient (Bio-Rad, Hercules, CA, USA) in a Protean IEF Cell (Bio-Rad) to determine the pI of proteins present in the extracted protein samples. For this, proteins were precipitated using a ReadyPrep 2-D starter kit (Bio-Rad) according to the manufacturer’s user guide. The recovered pellets were air-dried and resuspended in a sample solubilization solution (8 M urea, 50 mM DTT, 4% CHAPS, 0.2% carrier ampholytes, 3/10 Bio-LyteAmpholyte 40%). The focusing program used a stepwise approach, starting with 50 V for 12 h at 20 °C for active hydration of strips, conditioning strips at 250 V for 15 min, followed by a linear voltage increase at 4000 V for 2 h and final focusing at 24,000 V-h. After focusing, the IPG strips were applied to 12% SDS-PAGE, and after electrophoresis, the gels were stained as previously mentioned.

### 2.6. Techno-Functional Properties

To determine the solubility of protein extracts, 0.5 g of protein extracts was mixed with 5 mL water at different pH values (3, 5, 6.8, 9, and 11), which were adjusted with 0.1 M HCl or NaOH. The mixtures were stirred at 33 rpm for 30 min in a roller mixer at room temperature. Afterward, the slurries were centrifuged at 5000× *g* for 10 min at room temperature. The protein content in the supernatants was determined by the BCA method. The protein solubility (PS) was determined using the following equation (Equation (1)), where PCs represents the protein content in the supernatants and PCp represents the initial total protein for slurries:

(1) $$\left(\mathrm{PS}\;\%\right)=\frac{\mathrm{PCs}}{\mathrm{PCp}}\times 100$$

#### 2.6.1. Water and Oil Absorption

To determine water-holding capacity (WHC) or oil-holding capacity (OHC), a sample (0.5 g) of each protein extract was mixed with either water or olive oil (5 mL) in a test tube and stirred for 30 min at room temperature. The slurries were centrifuged at 5000× *g* for 10 min, and the supernatant and sediment were recovered. WHC or OHC were calculated based on weight gain using Equation (2).

(2) $$\mathrm{WHC}\;\mathrm{or}\;\mathrm{OHC}\;\left(g\;\mathrm{water}\;\mathrm{or}\frac{\mathrm{oil}}{g}\mathrm{protein}\;\mathrm{isolate}\right)=\frac{\mathrm{Ws}-\mathrm{Wds}}{\mathrm{Wi}}$$

where Ws represents the weight of the tube with sediment, Wds represents the weight of the tube with dry sample, and Wi represents the weight of the dry sample.

#### 2.6.2. Foaming Properties

Foaming properties were determined as reported previously [19], with some modifications. To determine foaming capacity (FC) protein extract solutions (0.5 mg/mL) were prepared using water at pH 5 or 6.8. The foaming process was achieved by sonication (Omni International, Omni Sonic Ruptor 400) for 30 s at 50% amplitude. The foaming capacity (FC) of the samples was evaluated based on the volume of the samples before (Vbs) and after (Vas) sonication, as shown in Equation (3).

(3) $$\left(\mathrm{FC}\;\%\right)=\frac{\mathrm{Vas}\;-\mathrm{Vbs}}{\mathrm{Vbs}\;}\times 100$$

The foam stability (FS) was determined by comparing the volume of foam after 30 min at room temperature (V30) and the volume of liquid in the samples (V) according to Equation (4).

(4) $$\left(\mathrm{FS}\%\right)=\frac{V30-V}{V\;}\times 100$$

#### 2.6.3. Emulsifying Properties

Emulsifying properties were determined as reported previously [19], with some modifications. Solutions with protein extracts (0.5 mg/mL) at pH 5 or 6.8 were mixed for 30 min at room temperature. Soybean oil was then added in a 40:60 ratio (slurry:oil), and the mixture was stirred for 2 min using a blade mixer until an emulsion was achieved. The volume of the third phase was measured, and the emulsifying capacity (EC) for each sample was determined considering the volume of the emulsion layer after stirring (Vas) and the volume of total liquid (Vbs), following Equation (5). The emulsion stability (ES) was evaluated by measuring Vas 30 min after emulsion formation at 80 °C.

(5) $$\left(\mathrm{EC}\;\%\right)=\frac{\mathrm{Vas}}{\mathrm{Vbs}}\times 100$$

### 2.7. Fourier Transform Infrared Spectroscopy (FT-IR)

Protein extracts were analyzed for their infrared spectra at pH 6.8 using a Bruker Vertex 70v Fourier transform infrared (FT-IR) spectrometer (Bremen, Germany) equipped with an attenuated total reflectance (ATR) accessory. Spectral measurements were recorded in the wavenumber range between 400 and 2500 cm^−1^, with a resolution of 4 cm^−1^. Spectrum acquisition of each sample was repeated three times using 180 scans for each protein. The secondary structure content of proteins was then determined by analyzing the amide band ranges from 1600 to 1700 cm^−1^ through second derivative analysis, as reported by Fidantsi and Doxastakis [20].

### 2.8. Statistical Analysis

All experiments were performed in triplicate. The results were expressed as the mean of three independent determinations through an analysis of variance (ANOVA). In a first experiment to obtain the tomato protein extracts from TSMH, a completely randomized design with a 3^2^ factorial arrangement was used, where the factors were molarity (0, 0.5, and 1) and pH (6.8, 8, and 10), generating 9 treatments with 3 replicates.

Results of the functional properties were analyzed using a completely randomized design with a factorial arrangement of 3^2^ (hexane and CO_2_; water and salt; and two pH values, namely 5 and 6.8). The significant differences between the means of treatments were determined by Tukey tests (*p* < 0.05) using SAS ^®^ System software (Ver.9.0).

## 3. Results

### 3.1. Fresh Tomato Pomace Yield and Chemical Composition

Upon analysis, it was found that 100 g of fresh tomatoes produced 7.4 g of pomace, 3.1 g of which were seeds, the remaining being pulp and peel, making up 41.9% of pomace. Therefore, it is expected that one ton of fresh tomatoes could generate 31 kg of seeds. Table 1 presents the chemical composition of tomato seeds. Seeds showed protein, fat, and raw fiber as major components, accounting 28.44%, 18.34%, and 26.19%, respectively.

### 3.2. Protein Extracts and SDS-PAGE Assays

**Figure 1:**
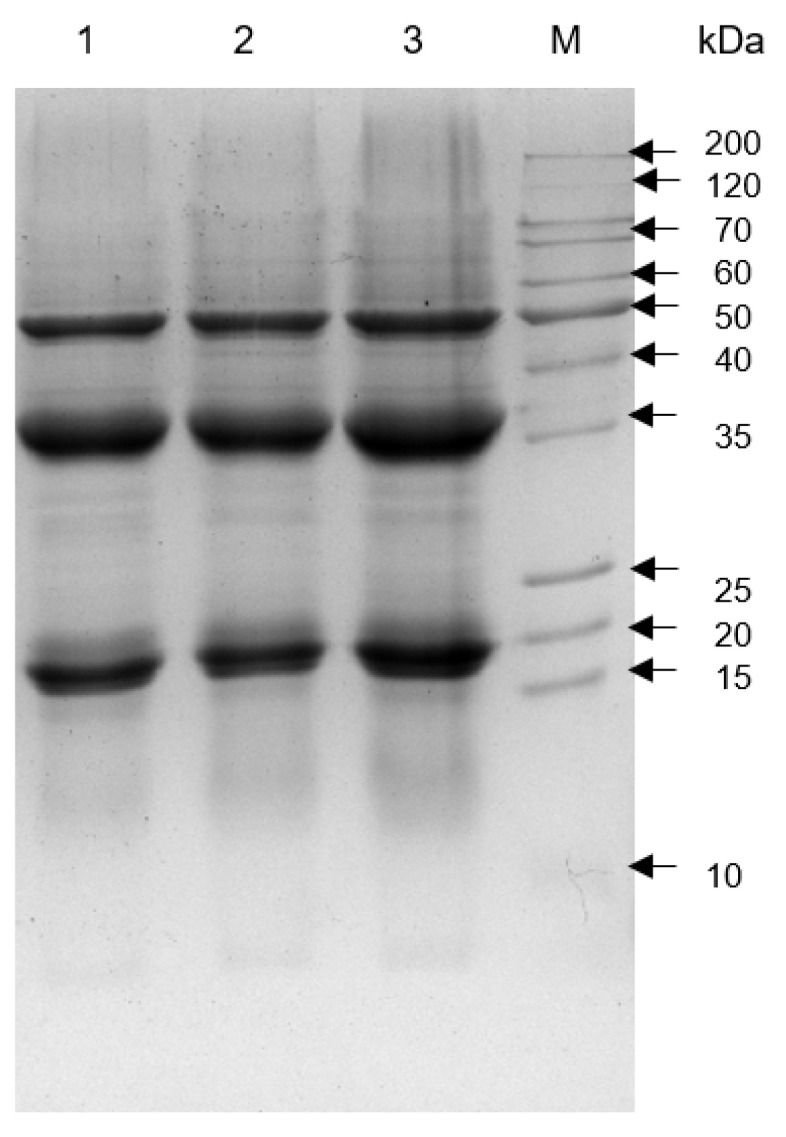
SDS-PAGE of total protein from tomato seed meals. Lanes: 1, undefatted tomato seed meal (TSM); 2, tomato seed meal defatted with hexane (TSMH); 3, tomato seed meal defatted with scCO_2_ (TSMC); M, molecular weight marker.

The electrophoretic patterns of tomato seed meal (TSM), TSMH (defatted with hexane), and TSMC (defatted with scCO_2_) are shown in Figure 1. SDS-PAGE showed 3 major high-intensity protein bands at 19, 35, and 50 kDa. Additionally, all tested meals exhibited proteins with molecular weights lower than 15 kDa and higher than 50 kDa.

**Figure 2:**
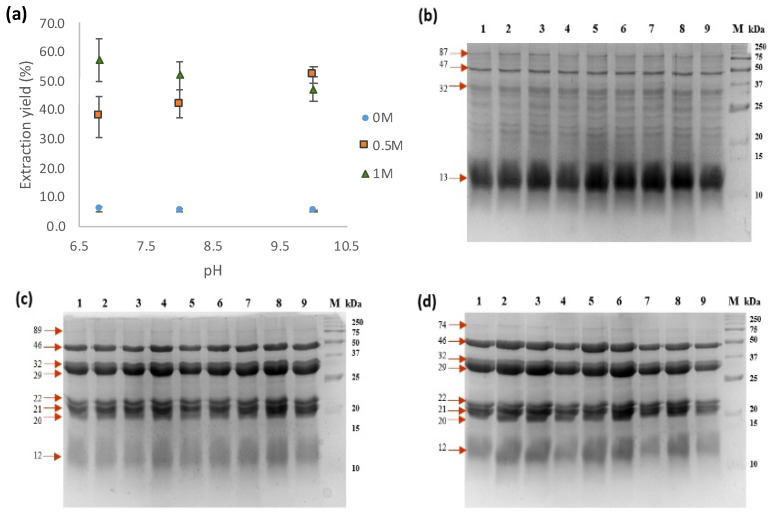
Protein extraction yield and electrophoretic patterns of extracts obtained using different solutions. (**a**) Extraction yields of protein extracts; (**b**) electrophoretic pattern profile of water-soluble proteins from tomato seed meal defatted with hexane (PEWH); (**c**) electrophoretic pattern profile of salt-soluble proteins from tomato seed meal defatted with hexane (PESH) (0.5 M NaCl); (**d**) electrophoretic pattern profile of salt-soluble proteins from tomato seed meal defatted with hexane (PESH) (1 M NaCl). Lanes: 1–3, pH 6.8; 4–6, pH 8; 7–9, pH 10; M, molecular weight marker.

In order to evaluate the impact of pH and solvent on the extraction of protein from meal defatted with hexane (TSMH), water and salt solutions (0.5 and 1 M NaCl) at pH values of 6.8, 8, and 10 were tested. The results showed that it is possible to obtain higher yields of proteins (37.7–57.2%) from meals using NaCl (Figure 2a). There was a significant difference (*p* < 0.05) between treatments, showing that NaCl improves the protein extraction yield. The use of 0.5 M NaCl resulted in an increase in extraction yield as pH increased (ranging from 37.7 to 52%). The tendency at 1 M NaCl was downwards; however, there was no statistical difference in the yield extraction with pH changes at this NaCl concentration (Figure 2a). No significant differences (*p* < 0.05) were observed using water as a solvent under different pH conditions, which showed lower extraction yields of approximately 5.5%. Protein extraction using salt solutions resulted in 8 to 10 times higher yields than extraction using water from tomato seed meal.

Analysis of the protein extracts obtained through SDS-PAGE demonstrated a similar protein pattern profile across various pH levels (6.8, 8, and 10). However, significant differences were observed in the protein profile when water or salt solutions were used for extraction (Figure 2b–d). Specifically, TSMH’s water-soluble protein extracts (PEWHs) displayed 13 protein bands ranging from 12 to 87 kDa, with a prominent 12 kDa band present in the extract. In contrast, TSMH’s salt-soluble protein extracts (PESHs) showed only five major protein bands ranging from 20 to 46 kDa (Figure 2c,d).

According to the findings, the most effective yield of PESH was achieved by utilizing a saline solution of 1 M NaCl at pH 6.8. Interestingly, the pH value did not appear to have a significant impact on protein extraction when using water. Consequently, the protein extracts from TSMC, PEWC, and PESC were obtained under comparable conditions. Their electrophoretic protein profiles showed 3 and 5 major proteins, ranging from 15 to 46 kDa (Figure S1).

### 3.3. Techno-Functional Properties

**Figure 3:**
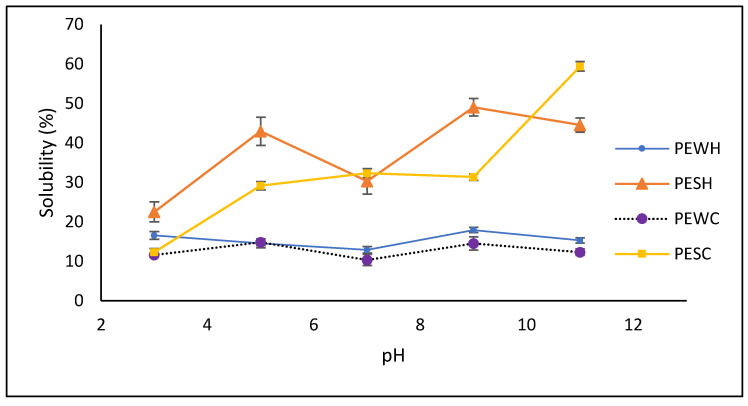
Solubility of obtained protein extracts. PESH and PESC: salt-soluble protein extracts treated with hexane and scCO_2_, respectively; PEWH and PEWC: water-soluble protein extracts treated with hexane and scCO_2_, respectively.

Protein solubility is an important factor in utilizing proteins for their techno-functional properties in foods. As depicted in Figure 3, protein extracts obtained from salt-soluble sources (PESH and PESC) exhibited greater solubility than those from water-soluble sources (PEWH and PEWC). Notably, PESH and PESC demonstrated optimal solubility (ranging from 31.6 to 59.9%) under alkaline conditions (pH 9–11). Interestingly, PESC showed no significant difference (*p* < 0.05) in solubility between pH levels ranging from 5 to 9, whereas PESH exhibited a typical V-shaped pattern and greater solubility at pH 5 and 9 (42.2% and 48.2%, respectively). Conversely, PEWH and PEWC displayed higher solubility at pH 9 (18%) and pH 5 (14.8%), while both samples exhibited lower solubility at pH 7.

As various commercial foods are formulated at pH 5–7, additional techno-functional properties were assessed under these specific pH conditions.

The defatting procedure had a significant effect (*p* < 0.05) on most functional properties, such as FC, EC, ES, and OHC (Table 2), but did not affect WHC values. Proteins extracted with scCO_2_ showed the best functional properties.

The extraction solution used for protein extraction also had a significant effect (*p* < 0.05) on most functional properties (Table 2). The FC and EC properties were best when proteins were extracted with water, whereas the WHC, OHC, and ES properties were best when proteins were extracted with saline solution.

pH also had a significant effect (*p* < 0.05) on the functional properties, with better values at pH 7.

The ability to bind water or oil in food products is crucial for modifying and enhancing their softness and mouthfeel. Table 3 displays the water-holding capacity (WHC) and oil-holding capacity (OHC) of extracted proteins. There was no significant difference in WHC or OHC at pH 5 between the PEWH, PESH, PEWC, and PESC protein extracts. At pH 5, both WHC and OHC values were lower than at pH 7 (Table 2), and all protein extracts demonstrated the ability to bind water and oil at pH 7. Notably, PEWC exhibited superior WHC compared to PEWH (1.3 and 0.5 g water/g protein extract, respectively). There was no significant difference (*p* < 0.05) in WHC between PESH and PESC at pH 7. OHC was higher than WHC for all protein extracts (Table 2 and Table 3), with both PEWC and PESC showing higher OHC values after being treated with scCO_2_.

Water protein extracts showed better foaming capacity (FC) than saline protein extracts (PESH and PESC). There was no significant difference (*p* < 0.05) in FC between pH 5 and pH 7 for PEWH. The results revealed that PEWH had FC values of 13.3% and 6.7% at pH 5 and pH 7, respectively (as shown in Table 3). PEWC had a similar FC of 10% at pH 5, but at pH 7, a higher FC of 72% was observed. However, all samples showed null foam stability (FS).

The majority of samples treated with hexane had a very low emulsifying capacity (EC), except for PESH, which showed a 17.8% EC at pH 7 (Table 3). On the other hand, the protein extracts treated with scCO_2_ exhibited different results. PEWC at pH 5 and 7, as well as PESC at pH 5, showed higher EC values (46.7% and 10%, respectively). However, the EC for PESC at pH 7 was 3.7 times lower (4.8%) than that of PESH under the same pH condition. It is worth noting that scCO_2_ treatment enabled stable emulsions for all protein extracts (PEWC and PESC), and PESC displayed better EC. At pH 7, the stability was the highest (96.3%) (Table 3).

### 3.4. Structural Analysis

**Figure 4:**
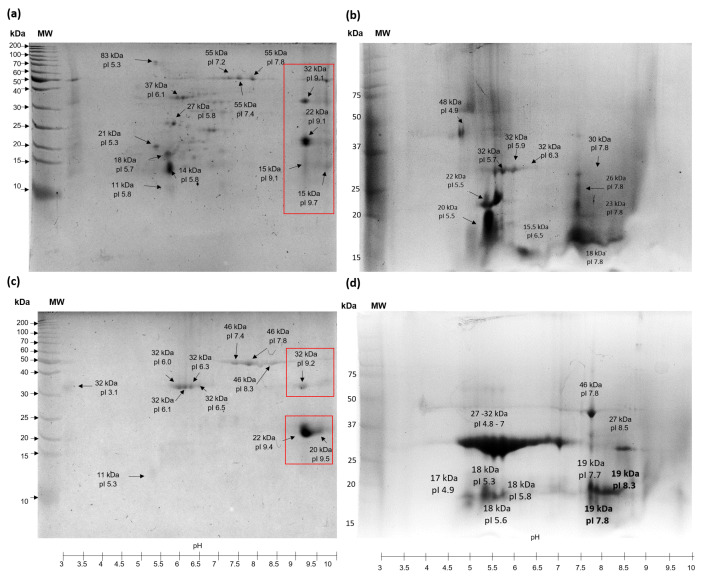
2D analysis of protein extracts from tomato seed meals. (**a**) PEWH, water–soluble protein from meal defatted with hexane; (**b**) PEWC, water–soluble protein extract from seed meal defatted with scCO_2_; (**c**) PESH, salt–soluble protein extract from seed meal defatted with hexane; (**d**) PESC, salt–soluble protein extract from seed meal defatted with scCO_2_. Squares show protein species hydrolyzed by scCO_2_ treatment.

Figure 4 displays the 2D analysis of protein extracts that are water-soluble (PEWH) and salt-soluble (PESH) from TSMH. The findings indicate that more protein species are present in PEWH compared to PESH. Additionally, it was observed that most protein species (approximately 70%) from PEWH have an isoelectric point (pI) ranging from pH 5 to 7.5. In contrast, PESH contains protein species with higher pI values, ranging from pH 7.4 to 9.5. The 2D analysis of PEWC (water-soluble protein extract treated by scCO_2_) revealed two regions in which protein species were grouped around pH 5 to 6.5 and 7.5. For PESC, almost 70% of protein species showed a pI around pH 4.8 to 7 (Figure 4b,d). Interestingly, protein extracts treated with scCO_2_ altered their 2D electrophoretic pattern, causing a displacement of proteins to acidic regions and revealing species with lower molecular weights. This is contrary to the extracts of hexane (PEWH and PESH) because scCO_2_ hydrolyzed some alkaline protein species (around pH 9 to 9.5), which can be observed in 2D gels of PEWH and PESH (squares in Figure 4a,c).

**Figure 5:**
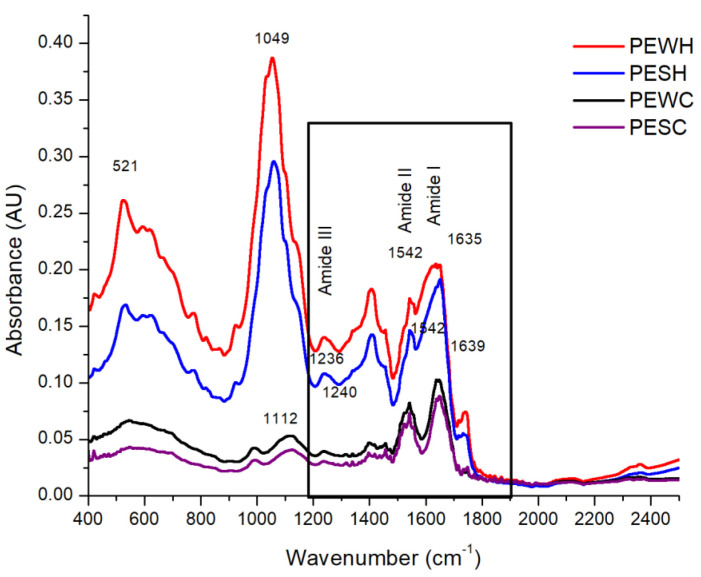
FT-IR spectra of water-soluble protein extract from tomato seed meal defatted with hexane (PEWH), salt–soluble protein extract from tomato seed meal defatted with hexane (PESH), water–soluble protein extract from tomato seed meal defatted with scCO_2_ (PEWC), and salt–soluble protein extract from tomato seed meal defatted with scCO_2_ (PESC). The numbers in the graph reference wavenumber.

FT-IR spectroscopy was used to analyze the main protein bands, which are reported between 1600 and 1700 cm^−1^, 1500 and 1600 cm^−1^, and 1200 and 1300 cm^−1^ for amide I, amide II, and amide III, respectively [21], although the presence of amide IV (625–767 cm^−1^) and amide V (537–606 cm^−1^) can also be noted [22]. Figure 5 shows the spectra obtained for PEWH, PESH, PEWC, and PESC.

The bands associated with amide I, amide II, and amide III are encircled by a solid continuous line and appear at 1635, 1542, and 1236 cm^−1^, respectively, for PEWH and PESH, while they can be found at 1639, 1542, and 1240 cm^−1^, respectively for PEWC and PESC. It is worth noting that the spectra of the samples extracted with hexane (PEWH and PESH) are similar to each other, besides showing a higher absorbance intensity. The spectra of the samples extracted with CO_2_ (PEWC and PESC) are also similar to each other but show a lower absorbance intensity when compared to the other samples. In addition to the absorbance intensity, a loss of signals in PEWC and PESC between 525 and 900 cm^−1^ was observed. Some of the vibrations located in this region are those emitted by amino acids such as tryptophan, tyrosine, threonine, and serine at 1000 to 1200 cm^−1^ [23]. A signal peak near 1000 cm^−1^ may mark the C-O-C vibration of the glycosidic bond or the C-O vibration. On the other hand, the peak near 628 cm^−1^ is characteristic of the out-of-plane O-H vibration [24]. C-N stretching vibrations can be assigned to wavelengths of 1444, 1438, 1369, 1354, 1166, 1142, 937, 746, and 525 cm^−1^ [25]. Signals at 501–523 cm^−1^ can be attributed to disulfide bonds, whereas the band at 1049 cm^−1^ can be attributed to S=O bonds and the signal at 1112 cm^−1^ to C-N bonds [26]. In this regard, as can be seen in Figure 5, both samples treated with scCO_2_ (PEWC and PESC) showed a lower absorbance intensity in the 521 cm^−1^ region. This could be attributed to the loss of disulfide bonds in the proteins present in PEWC and PESC as a result of the applied scCO_2_ treatment.

The amide I region (1600 to 1700 cm^−1^) is the principal absorption in proteins, serving as a marker for secondary protein structure due to the stretching vibrations of the C=O and C-N groups of the protein backbone chain [27]. Accordingly, FT-IR spectra were analyzed using the data collected and deconvolved in the amide I region (Figure S2). As listed in Table 4, all protein extracts possess significant β-sheet secondary structure content (ranging from 33.2 to 48.7%). PEWH displays lower helix content (19.2%) and more turn content (31.7%) than PEWC (24.4% and 25.3%, respectively). In contrast, PESH shows higher helix content (8.0%) and lower unordered content (11.3%) than PESC, which exhibits null helix and 21.1% unordered content. Therefore, scCO_2_ has an impact on the helix secondary structure content of albumins and globulins obtained from tomato seeds. For albumins, scCO_2_ increases helix content, while for globulins, it reduces helix content.

## 4. Discussion

Different studies have indicated that the manufacturing of tomato paste results in between 70 and 75 Kg of solid waste per ton of fresh tomatoes. Seeds account for approximately 60% of this waste (approximately 42 kg) [28]. Additionally, Sarkar and Kaul [29] reported that a pilot plant generated waste pomace consisting of 26.2% peel and 73.8% seeds. However, the results obtained in this study are lower than those reported in both abovementioned studies, possibly due to differences in tomato varieties, growing conditions, and cultivation region [4,30].

### 4.1. Protein Extracts and SDS-PAGE Assays

In this study, the protein content of used seeds was similar to that previously reported [29,31]. Protein content from tomato seeds was higher than some cereals, like corn, rice, barley, sorghum, rye, and millet (ranging from 6.7 to 19.4%) [32]. Furthermore, the protein content of tomato seeds was comparable to that of certain legumes, such as chickpeas, lentils, and peas (ranging from 21.2 to 32.9%) [33,34]. From Figure 1, it can be seen that there is no difference in the protein profiles between the TSM, TSMH, and TSMC, indicating that the the oil extraction process did not affect the obtained proteins.

The use of NaCl resulted in a significantly higher protein extraction content (*p* < 0.05), suggesting a higher proportion of globulins in the tomato seed flour compared to albumins. These findings are consistent with previous reports [35]. The improved results with NaCl may be attributed to the chloride ions, which increase solubility by electrostatic repulsion when binding to positively charged protein groups [36]. Moreover, alkaline conditions promote the acquisition of net-negative charges by proteins, facilitating molecule repulsion and thereby enhancing protein solubility at 0.5 M NaCl [37]; at a concentration of 1M NaCl, there are probably enough ionic interactions between the salt and the proteins to extract most of the proteins in the meal, minimizing the pH effect.

Similar results using alkaline conditions for protein extraction from tomato seeds were reported by Liadakis et al. [31], who achieved a yield of 43.6% at pH 11.5 and at 50 °C, while Shao et al. [38] reported yields between 37.5 and 45.41% at pH values of 9 to 13. In the first paper [31], they used high-temperature extraction at 50 °C, while in this work, room temperature extraction was used. In the second paper [38], the process was first carried out at high pH (9 to 11), followed by isoelectric precipitation, whereas in the present work, solely extraction was used, and protein recovery was achieved through centrifugation.

SDS-PAGE shows the pattern profiles of proteins in a sample. The electrophoretic pattern obtained from tomato seed meal is similar to that reported by Mechmeche et al. [39], who obtained protein with three major bands between 15 and 50 kDa from tomato seeds for bacterial fermentation. However, PEWH showed 11 additional protein bands in comparison with the results of Sogi et al. [35], who reported only two protein bands with molecular weights of about 19 and 27 kDa extracted using water. This difference could be attributed to the acrylamide concentration used in the electrophoresis gel, as we used a 14% acrylamide gel, and Sogi et al. [35] used a 10% acrylamide gel. The protein profile of salt-soluble protein extract treated with hexane (PESH) closely resembled that of the oil–protein solution interface of tomato seed globulins analyzed by Sarkar et al. [30]. Sarkar et al. reported the presence of five major protein bands ranging from 10 to 48 kDa. However, Figure 2b,c reveal two additional bands (22 and 35 kDa). This difference in bands may be attributed to the process used after extraction with NaCl 1 M, whereby Sarkar et al. precipitated proteins at a pH of 3.5. It is possible that the bands at 22 and 35 kDa did not precipitate at that pH and, thus, were not present in the protein isolate reported by Sarkar et al. [40].

### 4.2. Techno-Functional Properties and Structures of Some Proteins Were Affected by scCO_2_ Treatment

The techno-functional properties of proteins play a crucial role in producing different food items because they allow proteins to function as foaming, emulsifying, or gelling agents, which stabilize the food system. Protein solubility is essential for proteins to develop their techno-functional applications in different foods. Factors such as the composition, sequence, and type of amino acids on the protein surface influence protein solubility. Extrinsic factors such as ionic strength, temperature, pH, and other components in the solvent also affect the interactions, structure, and conformation of proteins [41]. The lower solubility of protein extracts in the pH range of 4 to 7 is due to the majority of protein species having a pI close to this pH range (Figure 4), resulting in protein–protein interactions and further insolubility [36]. It has been reported that scCO_2_ can promote changes in interfacial and surface properties, leading to a partially unfolded structure of proteins known as a molten globule, which is characterized by a somewhat compact structure and a high degree of hydration and side-chain flexibility, with the exposure of buried hydrophobic residues. Additionally, the molten globule state retains a significant amount of native secondary structure but exhibits limited tertiary folds [42,43]. In this sense, the lower solubility of PESC at pH 5 to 9 could be promoted by the molten globule state adopted by different protein species in the extract. When the pH conditions are changed, the exposed hydrophobic residues interact with others, leading to the formation of protein aggregates.

Water-holding capacity (WHC) and oil-holding capacity (OHC) are important properties to maintain desired texture characteristics in different foods. A high WHC or OHC can help reduce the loss of humidity or oil during food processing or storage, leading to improved softness, mouthfeel, and sensory acceptability, among other effects. In a previous study, Maldonado-Torres et al. [2] reported higher WHC values from tomato seed meal (3.18–4.31 g water/g flour) and lower OHC values (2–2.3 g oil/g flour) than those obtained in this study. This difference can be attributed to the presence of carbohydrates, which have hydrophilic regions, and hydrophilic amino acids of proteins, which could interact and bind more water molecules [44]. Also, a lower WHC was obtained in this study in comparison with the WHC (4.95 g water/g protein isolate) of tomato seed protein isolate. In this study, the OHC measured at pH 7 exceeded the value of 1.18 g oil/g protein isolate obtained by Özyurt et al. [45], although it was similar to the OHC obtained at pH 5 in this work. The difference in WHC and OHC between the extracts at pH 5 and 7 may be attributed to the presence of different proteins with varying isoelectric points within the extracts, as depicted in Figure 4a–d. There are several proteins with isoelectric points close to 5, so if they are in a medium range with a pH of 5 (very close to their isoelectric point), their solubility decreases, and their conformation may also change; therefore, these properties change, as it has been reported that altering the pH conditions of proteins can lead to a change in their conformation [46,47]. Notably, the OHC improvement observed at pH 7 was between four and five times higher than the value reported by Özyurt et al. [45] in protein extracts obtained by scCO_2_ (PEWC and PESC). The greater OHC suggests the presence of higher levels of non-polar amino acids exposed on the surface of extracts, allowing for better retention of oil [48], which is a consequence of structural changes promoted by scCO_2_. The results obtained for OHC (6.2 g oil/g protein extract) in PESC samples were higher than those reported by Kheto et al. [49] for guar germ proteins. Based on these results, it is suggested that PEWC, PESH, and PESC could be used as thickeners for sauces or soups. This is because WHC levels ranging from 1.97 to 4.72 g water/g sample are sufficient to maintain adequate viscosity in these food systems [50]. Additionally, PESH, PEWC, and PESC could be utilized to design or improve food products that are rich in lipids, such as meat formulations or bakery products. This is due to their OHC properties, which can help to retain flavor, maintain texture and mouthfeel, and reduce rancidity [51].

It is known that foams are formed when proteins are unfolded and grouped together to form a layer at the boundary between the aqueous and air phases, which helps to maintain air bubbles and prevent their collapse. The solution used for protein extraction had a significant effect (*p* < 0.05) on the vast majority of functional properties, with higher FC and EC values in the water-extracted proteins. This could be due to the flexibility of the molecules and the higher proportion of low-molecular-weight proteins in the water-extracted proteins (Figure 2b,c), which could encapsulate more air bubbles due to their rapid diffusion on the air–water surface. This is in agreement with the observations reported by Tang et al. (2023) [52]. Interestingly, PEWC at pH 7 showed almost 12 times higher FC, even though all extracts showed null foam stability (<2 min). To this respect, there are reports of proteins without foam stability for a rapeseed protein isolate obtained with a weakly acidic salt extraction and ultrasound (Zhang et al., 2024) [53], and the same is reported by [54] for proteins from an insect hydrolyzed with various enzymes.

Some studies suggest that higher protein concentrations (1% *w*/*w*) can improve foaming capacity and stability (FS) by enabling faster adsorption and more uniform bubble sizes [55]. Therefore, further studies on increasing protein concentrations would be highly warranted to assess the resulting FC and FS.

Higher values were observed for the emulsification capacity (EC) of protein extracts obtained through scCO_2_. This difference may be attributed to the structural or conformational rearrangements that scCO_2_ promotes in the protein species of PEWC and PESC, which modify the hydrophilic–hydrophobic balance of proteins on the surface. This improvement in orientation of hydrophilic and lipophilic amino acids allows for better emulsifying activity [56]. Other studies have reported that tomato protein extracts show better EC and ES (ranging from 40–80%) under alkaline conditions (pH 8–10), which could be attributed to the greater solubility achieved under these pH conditions. This, in turn, allows for the maintenance of a better hydrophilic–hydrophobic balance and structural conformations that lead to increased emulsifying activity [38,45]. On the other hand, higher ES values were observed in saline-extracted proteins (*p* < 0.05). This could be related to the higher proportion of beta structure in these proteins (Table 4). Tang et al. (2023) [52] reported something similar in a soy protein isolate at a pH of 9.

The improvement in the properties of PEWC and PESC, including OHC, FC, EC, and ES, could be attributed to structural modifications and conformational changes promoted by scCO_2_ treatment, which changes their interfacial properties. Additionally, scCO_2_ treatment was found to hydrolyze alkaline proteins in PEWC and PESC, as evidenced by 2D electrophoresis of proteins with a pI of pH 9. Similar results were reported by Dunford et al. [57], who observed that moisture content can induce changes in the structure of sarcoplasmic proteins from Atlantic mackerel treated with scCO_2_. They reported that protein aggregation led to an increase in the molecular-weight of protein species in the extracts. Hydrolysis is another mechanism by which structural changes can occur in proteins, and CO_2_ accelerates this reaction by facilitating proton addition, which is a crucial step in the reaction [58,59]. Also, scCO_2_ can promote the unfolding and exposure of the buried internal sulfhydryl groups and even disrupt the disulfide bonds [60], as mentioned above and observed in Figure 5. In addition, the hydrolysis observed in this study due to the application of scCO_2_ could be attributed to the presence of water in the sample. This water, under subcritical conditions (as employed with scCO_2_), generates the formation of hydronium ions (H_3_O^+^) and hydroxide ions (OH^−^), which have the ability to act as acidic or basic catalysts, promoting the hydrolysis of proteins [61,62].

Protein conformational changes can be explained by various factors, such as the presence of scCO_2_ and H_2_O, which can lead to the formation of carbonic acid and decreases in pH levels. As a result, alterations in environmental conditions can impact the three-dimensional structures of proteins, although some may recover their structure, with only minor changes observed [58]. In this study, the moisture content in the raw material and the scCO_2_ treatment were shown to modify the secondary structure content of PEWH and PESC, as evidenced by FT-IR spectroscopy. Additionally, experimental assays revealed that scCO_2_ can alter the amounts of helices, sheets, and random coils in proteins, leading to enhanced techno-functional properties that make whey proteins more applicable in the food industry [43,63].

The protein extracts obtained in this study mostly consisted of sheet structures. These findings align with the results reported by Ma et al. [64], who also obtained protein extracts from cottonseed meal using various pretreatments. The scCO_2_ treatment had an impact on the helical structure content, with higher helical content observed for PEWC and lower helical content observed for PESC, leading to an increase in turns and unordered structure, respectively. It is common for the secondary structure of globular proteins to be altered due to changes in helical content [22]. Lima et al. [65] reported a decrease in the helical content structure of α-lactalbumin when treated with scCO_2_.

## 5. Conclusions

Supercritical fluid extraction using CO_2_ (scCO_2_) is an emerging technology with numerous advantages. It is a chemical-free process that is safe to operate and can offer an alternative to biomass processing. This technology can be used to create new products or make use of agroindustrial waste, which, in turn, can be used to design new food products or enhance existing ones. In this study, scCO_2_ treatment was used to defat tomato seed protein extracts, which improved their techno-functional properties. The treatment resulted in significant structural changes and modified interfacial properties of the protein extracts, with changes in helical content observed. These findings demonstrate the potential of scCO_2_ treatment to produce modified proteins with practical applications in the food industry.

The improvement or compromise of protein techno-functional and technological applications depends on the structural or conformational changes produced by the applied method to the protein. To modify the structure or conformation, protein hydrolysis presents a viable alternative. This study suggests that scCO_2_ treatment may serve as a physical method for hydrolysis. Further research is required to explore the mechanism and conditions of hydrolysis. However, scCO_2_ may prove to be a promising option for industrial protein hydrolysis applications.

## Figures and Tables

**Table 1:** Chemical composition of tomato seed meal.

Component	g/100 g Tomato Seed Meal
Protein	28.44 ± 0.19
Fat	18.34 ± 0.21
Moisture	8.18 ± 0.26
Crude fiber	26.19 ± 1.36
Ash	3.9 ± 0.14
* Carbohydrates	14.95 *

* Value obtained by difference. Number of replications: 3.

**Table 2:** Analysis of the effects of factors on the techno-functional properties of protein extracts from tomato seed meal.

Factor	FC	EC	WHC	OHC	ES
**Defatting**					
CO_2_	21.0 ± 9.0 A	27.0 ± 5.9 A	0.9 ± 0.2 A	3.6 ± 0.6 A	43.7 ± 10.8 A
Hex	5.4 ± 1.7 B	5.1 ± 2.2 B	0.8 ± 0.3 A	2.9 ± 0.5 B	0.9 ± 0.02 B
**Protein extraction**					
Salt	0.9 ± 0.02 B	8.4 ± 1.9 B	1.2 ± 0.3 A	3.8 ± 0.7 A	38.7 ± 12.3 A
Water	25.6 ± 8.2 A	23.8 ± 6.9 A	0.5 ± 0.1 B	2.8 ± 0.5 B	6.0 ± 1.5 B
**pH**					
pH 5	6.3 ± 1.8 B	14.6 ± 5.7 B	0.2 ± 0.02 B	1.4 ± 0.05 B	17.4 ± 7.1 B
pH 7	20.1 ± 9.1 A	17.6 ± 5.4 A	1.5 ± 0.2 A	5.1 ± 0.4 A	27.2 ± 12.1 A
Significance					
Defatting	**	**	NS	**	**
Protein extraction	**	**	**	**	**
pH	**	**	**	**	**

Means (±std. error). The use of different uppercase letters within each factor (defatting, protein extraction, and pH) within each response variable (FC, EC, WHC, OHC, and ES) indicates significant differences (*p* < 0.05). **: (*p* < 0.01); NS: not significant; *n* = 12.

**Table 3:** Techno-functional properties of tomato protein extracts from TSMH and TSMC (defatted with hexane and CO_2_, respectively).

Property	TSMH				TSMC			
	PEWH_pH5_	PEWH_pH7_	PESH_pH5_	PESH_pH7_	PEWC_pH5_	PEWC_pH7_	PESC_pH5_	PESC_pH7_
**WHC** *	0.3 ± 0.0C	0.5 ± 0.1C	0.2 ± 0.03C	2.4 ± 0.3A	0.3 ± 0.0C	1.2 ± 0.07B	0.2 ± 0.0C	2.0 ± 0.1A
**OHC** ^+^	1.3 ± 0.05C	3.2 ± 0.5B	1.5 ± 0.05C	5.9 ± 0.5A	1.3 ± 0.0C	5.2 ± 0.4A	1.6 ± 0.1BC	6.2 ± 0.3A
**FC** (%) ^«^	13.3 ± 3.3B	6.7 ± 1.6BC	0.9 ± 0.06C	0.9 ± 0.05C	10.0 ± 0.0B	72.2 ± 2.7A	0.9 ± 0.05C	0.9 ± 0.03C
**EC** (%) ^α^	0.9 ± 0.03D	0.9 ± 0.05D	0.9 ± 0.03D	17.8 ± 1.1B	46.7 ± 3.3A	46.7 ± 1.6A	10.0 ± 0.0C	4.8 ± 0.2CD
**ES** (%) ^β^	0.9 ± 0.02C	0.9 ± 0.05C	0.9 ± 0.05C	0.9 ± 0.05C	11.3 ± 1.2C	10.7 ± 0.3C	56.7 ± 6.6B	96.3 ± 3.7A

* WHC, water-holding capacity (g water/g protein extract); ^+^ *OHC*, oil-holding capacity (g oil/g protein extract); ^«^ *FC*, foaming capacity; ^α^ *EC*, emulsifying capacity; ^β^ *ES*, emulsion stability after 30 min at 80 °C. Mean of triplicate assays (±std. error). Values with different letters within rows indicate significant difference (*p* < 0.05). TSMH: tomato seed meal defatted with hexane; PEWH: water-soluble protein extract from tomato seed meal defatted with hexane; PESH: salt-soluble protein extract from tomato seed meal defatted with hexane; TSMC: tomato seed meal defatted with scCO_2_; PEWC: water-soluble protein extract from tomato seed meal defatted with scCO_2_; PESC: salt-soluble protein extract from tomato seed meal defatted with scCO_2_. Different uppercase letters within each row indicate significant differences (*p* < 0.05).

**Table 4:** Secondary structure content in the protein extracts.

Secondary Structure	TSMH		TSMC	
	PEWH	PESH	PEWC	PESC
**Helix (%)**	19.2 ± 1.3	8.0 ± 1.2	24.4 ± 1.8	NS
**Sheet (%)**	33.2 ± 1.5	48.7 ± 1.4	33.5 ± 1.1	48.5 ± 1.4
**Turns (%)**	31.7 ± 1.9	32.0 ± 2.1	25.3 ± 1.6	30.4 ± 1.3
**Unordered (%)**	15.9 ± 1.6	11.3 ± 1.7	16.8 ± 1.1	21.1 ± 1.4

NS: not shown; number of replications: 3; PEWH, water-soluble protein defatted with hexane; PEWC, water-soluble protein extract defatted with scCO_2_; PESH, salt-soluble protein extract defatted with hexane; PESC, salt-soluble protein extract defatted with scCO_2_. TSMH, tomato seed meal defatted with hexane; TSMC, tomato seed meal defatted with scCO_2._

## Data Availability

The original contributions presented in the study are included in the article/Supplementary Materials, further inquiries can be directed to the corresponding author.

## Supplementary Materials

The following supporting information can be downloaded at: https://www.mdpi.com/article/10.3390/foods13071045/s1, Figure S1. SDS-PAGE of protein extracts from tomato seed meals defatted with scCO_2_. Figure S2. Deconvolved FT-IR spectra of protein extracts from tomato seed meals treated with hexane and scCO_2_.
