# Supplementary material for: Supercritical CO2 Treatment to Modify Techno-Functional Properties of Proteins Extracted from Tomato Seeds
Source: Foods. 2024 Mar 28;13(7):1045. doi: 10.3390/foods13071045 (PMC11011313; doi:10.3390/foods13071045)
Supplement: Supplementary file 1 [file foods-13-01045-s001.zip › foods-2919110-supplementary.pdf]

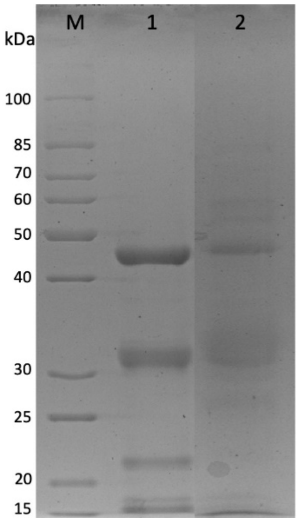

Figure S1. SDS-PAGE of protein extracts from tomato seed meals defatted with scCO2. Lanes: M, molecular weight marker; 1, Proteins extracted with 1M NaCl; 2, Proteins extracted with water.

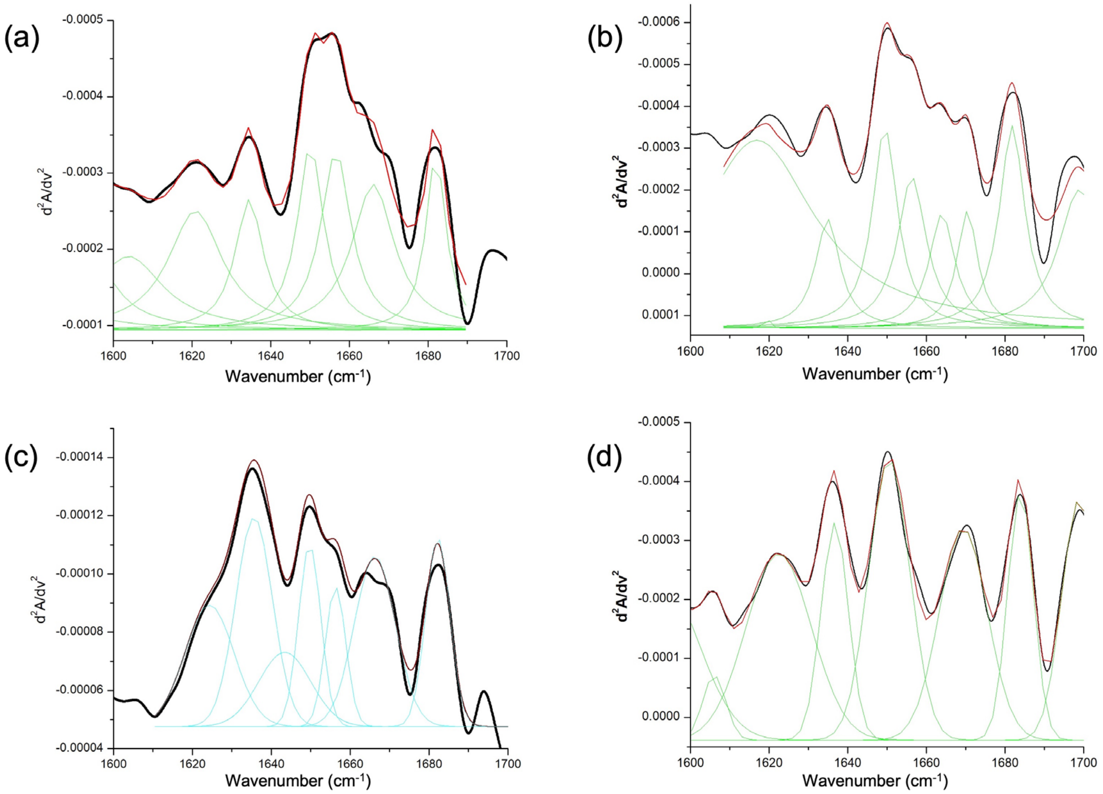

Figure S2. Deconvolved FT-IR spectra of protein extracts from tomato seed meals treated with hexane and $scCO_2$. a) PEWH, Proteins extracted with water from tomato seed meal

defatted using hexane b) PEWC, Proteins extracted with water from tomato seed meal defatted using supercritical CO_2; c) PESH, Proteins extracted with 1M NaCl from tomato seed meal defatted using hexane; d) PESC, Proteins extracted with 1M NaCl from tomato seed meal defatted using supercritical CO_2.
